# Infiltration of the synovial membrane with macrophage subsets and polymorphonuclear cells reflects global disease activity in spondyloarthropathy

**DOI:** 10.1186/ar1501

**Published:** 2005-01-21

**Authors:** Dominique Baeten, Elli Kruithof, Leen De Rycke, Anemieke M Boots, Herman Mielants, Eric M Veys, Filip De Keyser

**Affiliations:** 1 Department of Rheumatology, Ghent University Hospital, Ghent, Belgium; 2 Department of Pharmacology, Organon NV, Oss, The Netherlands

**Keywords:** disease activity, histopathology, spondyloarthropathy, surrogate marker, synovium

## Abstract

Considering the relation between synovial inflammation and global disease activity in rheumatoid arthritis (RA) and the distinct but heterogeneous histology of spondyloarthropathy (SpA) synovitis, the present study analyzed whether histopathological features of synovium reflect specific phenotypes and/or global disease activity in SpA. Synovial biopsies obtained from 99 SpA and 86 RA patients with active knee synovitis were analyzed for 15 histological and immunohistochemical markers. Correlations with swollen joint count, serum C-reactive protein concentrations, and erythrocyte sedimentation rate were analyzed using classical and multiparameter statistics. SpA synovitis was characterized by higher vascularity and infiltration with CD163^+ ^macrophages and polymorphonuclear leukocytes (PMNs) and by lower values for lining-layer hyperplasia, lymphoid aggregates, CD1a^+ ^cells, intracellular citrullinated proteins, and MHC–HC gp39 complexes than RA synovitis. Unsupervised clustering of the SpA samples based on synovial features identified two separate clusters that both contained different SpA subtypes but were significantly differentiated by concentration of C-reactive protein and erythrocyte sedimentation rate. Global disease activity in SpA correlated significantly with lining-layer hyperplasia as well as with inflammatory infiltration with macrophages, especially the CD163^+ ^subset, and with PMNs. Accordingly, supervised clustering using these synovial parameters identified a cluster of 20 SpA patients with significantly higher disease activity, and this finding was confirmed in an independent SpA cohort. However, multiparameter models based on synovial histopathology were relatively poor predictors of disease activity in individual patients. In conclusion, these data indicate that inflammatory infiltration of the synovium with CD163^+ ^macrophages and PMNs as well as lining-layer hyperplasia reflect global disease activity in SpA, independently of the SpA subtype. These data support a prominent role for innate immune cells in SpA synovitis and warrant further evaluation of synovial histopathology as a surrogate marker in early-phase therapeutic trials in SpA.

## Introduction

Whereas classical analysis of synovial tissue in chronic inflammatory arthritis suggested that synovitis is a nonspecific phenomenon, a number of studies using new molecular tools and synovial biopsies obtained during active disease indicated clear histopathological differences between spondyloarthropathy (SpA) and rheumatoid arthritis (RA), which are the two most frequent forms of chronic autoimmune arthritis [1-4]. These differences were explored as a diagnostic tool in undifferentiated arthritis [5,6], and highly disease-specific markers as well as less pronounced synovial features turned out to be useful in multiparameter classification models [7,8]. Since some of these features are pathophysiologically related to specific disease mechanisms [3,4], it is tempting to hypothesize that histopathological characteristics of inflamed synovium directly reflect the disease process.

In RA, it has been shown that synovial features may help to distinguish different subgroups corresponding to distinct pathogenetic mechanisms and clinical phenotypes. Indeed, three main histological types of synovitis can be distinguished in RA: one type characterized by follicular organization with high numbers of B cells and plasma cells, another type with diffuse infiltration by essentially T lymphocytes and macrophages, and a third, granulomatous type, which is less frequent [9]. The different histological types are stable over time within one individual, are linked with different cellular and molecular disease pathways, as evidenced, for example, by abundant IL-10 in the follicular type, and are related to phenotypic differences such as seronegativity in the diffuse type and extra-articular manifestations in the granulomatous type [10,11]. Besides distinguishing subtypes within one disease, some of these features may also reflect global disease activity and thus be valuable candidates for evaluation as surrogate markers in trials of new, targeted therapies in autoimmune arthritis. Synovial macrophages have been shown to be related to scores for local disease activity as well as to articular damage in RA [12,13]. Sublining macrophages, but also T cells and plasma cells, were increased in clinically involved versus clinically uninvolved knee joints [14], while sublining macrophages were also increased in joints in active RA compared with joints in end-stage disease [15]. Taken together, these various findings strongly suggest that the number of sublining macrophages is a good reflection of active disease processes in RA. This interpretation was further validated by demonstrating that synovial sublining macrophages can be used as surrogate marker for global disease activity in clinical trials evaluating antirheumatic therapy in RA [16].

In contrast with the situation with RA, these issues have not yet been fully assessed in SpA. We have previously demonstrated a correlation between synovial histology and local disease activity in SpA [1] and the absence of manifest differences in synovial histopathology between psoriatic arthritis (PsA) and other SpA subtypes. Considering the data in RA synovitis, the increase of specific macrophage subsets and polymorphonuclear leukocytes (PMNs) in SpA synovium compared with RA [2], and the strong and rapid reduction of synovial macrophages, T lymphocytes, and PMNs during treatment with anti-tumor-necrosis-factor (TNF)-α in SpA [17,18], the objective of the present study was to analyze in more detail whether histopathological features of the synovial membrane reflect specific phenotypes and/or global disease activity in SpA.

## Materials and methods

### Patients and samples

The study included 99 SpA patients fulfilling the criteria of the European Spondyloarthropathy Study Group [19]. One cohort consisted of 82 patients, including 19 with ankylosing spondylitis (AS), 33 with PsA, 24 with undifferentiated SpA (USpA), 4 with SpA associated with inflammatory bowel disease, and 2 with reactive arthritis. Since we had previously found no major differences between these SpA subgroups for the synovial histopathology markers used in the present study, we considered them collectively as having SpA (unpublished data). All patients had active disease at the time of inclusion, as evidenced by a mean swollen joint count (SJC) of 3.5 ± 4.1 (mean ± standard deviation), a mean serum C-reactive protein (CRP) concentration of 33 ± 45 mg/L, and a mean erythrocyte sedimentation rate (ESR) of 28 ± 24 mm/hour. The mean duration of disease was 5.5 ± 5.4 years, and 23 of the 82 patients were being treated with a disease-modifying antirheumatic drug (DMARD); none of the patients were being treated with corticosteroids. All patients had at least one swollen knee joint, from which synovial biopsies were sampled by needle arthroscopy.

As an independent validation group, a second cohort of 17 SpA patients (4 with AS, 5 with PsA, 8 with USpA) fulfilling the same inclusion criteria was included in the study. This group had a mean SJC of 5.5 ± 5.4, a mean serum CRP of 38 ± 48 mg/L, and a mean ESR of 31 ± 27 mm/hour. None of these patients was receiving DMARDs or corticosteroids. The mean duration of disease in this group was 10.8 ± 10.2 years.

For the control group, we included 86 patients fulfilling the American College of Rheumatology criteria for RA [20]. As for the SpA cohort, all RA patients had at least one swollen knee joint and had active disease, with a mean SJC of 9.2 ± 6.6, a mean serum CRP of 58 ± 67 mg/L, and a mean ESR of 41 ± 27 mm/hour. The mean duration of disease was 6.0 ± 7.5 years. Fourteen patients were receiving DMARDs, 5 patients were receiving corticosteroids, and 21 patients were receiving both.

In all the patients, synovial tissue biopsies (16 from each person) were obtained by needle arthroscopy of a clinically involved knee joint, as described previously [21]. All patients gave their written, informed consent before inclusion in the study, which was approved by the Ethics Committee of the Ghent University Hospital.

### Synovial histopathology

Synovial biopsies were fixed, stained, and scored as described previously [1-4]. Briefly, eight paraffin-embedded biopsies were stained with H&E for histological analysis, including mean thickness of the synovial lining-layer, vascularity of the sublining layer, infiltration of the sublining layer, and the presence of lymphoid aggregates, plasma cells, and PMNs. A separate analysis in 93 samples showed that the evaluation of the number of blood vessels and the number of plasma cells on H&E staining correlated well with staining for, respectively, the endothelial marker CD146 (*r *= 0.436; *P *< 0.0001) and the plasma cell marker CD138 (*r *= 0.621; *P *< 0.0001). The remaining eight biopsies were embedded in tissue-freezing medium and used for immunohistochemistry with the following antibodies: anti-CD1a (interdigitating dendritic cells, mouse, monoclonal; Dako, Glostrup, Denmark), anti-CD3 (T cells, mouse, monoclonal; Dako), anti-CD20 (B cells, mouse, monoclonal; Dako), anti-CD68 (monocytes and macrophages, mouse, monoclonal, clone EBM11; Dako), anti-CD163 (scavenger receptor expressed on mature tissue macrophages, mouse, monoclonal, clone Ber-MAC3; Dako), anti-L-citrulline (citrullinated peptides, rabbit, polyclonal; Biogenesis, Poole, UK), and mAb 12A (detecting MHC class II–HC gp39 peptide complexes, mouse, monoclonal; NV Organon, Oss, Netherlands). Parallel sections were stained with irrelevant origin-, isotype-, and concentration-matched antibody as negative control. Stained sections were coded and analyzed by two independent observers, who were blinded to the diagnosis and clinical data. Due to the low number of positive cells in each sample for anticitrulline, anti-CD1a, and mAb 12A staining, these parameters as well as lymphoid aggregates were scored as present or absent. For all other parameters, the analysis included all areas of the biopsies, and a global score was given for each parameter, using a semiquantitative 4-point scale: 0 represented the lowest and 3 the highest level of expression [1-4]. As some histological markers are more abundant than others, the scoring system was calibrated for each marker separately by examining a representative number of samples. In case of discordant scores between the two observers, the mean of the two scores was used. Since anti-CD68 and anti-CD163 staining, which recognizes a particular subset of the CD68^+ ^macrophages [2], was observed in both the synovial lining layer and the synovial sublining layer, these markers were scored separately in the two compartments. An overview of the 15 synovial parameters is given in Table 1.

### Statistics

The histopathological features of the synovial membrane in SpA and RA were compared using the Mann–Whitney *U *test for semiquantitative parameters and the χ^2 ^test for dichotomous parameters. Histopathological subgroups were identified by clustering analysis (within-group average linkage with Pearson correlation) using SPSS version 12.0 software (SSPS Inc, Chicago, IL, USA). Correlations between semiquantitative histological parameters and clinical disease activity markers (SJC, CRP, ESR) were calculated using the Spearman ρ test. For dichotomous histological markers, the clincal disease activity parameters of the positive and negative groups were compared using an unpaired Student's *t*-test. A *P *value of less than 0.05 was considered statistically significant.

The relation between the 15 histological parameters and the 3 clinical parameters was also analyzed by SAM (significance analysis of microarray) software, a statistical analysis model that was specifically developed for multiparameter datasets [22] (see also ). Measuring the relation between changes in the input parameter (which are here the 15 histological features) and changes in the response variable (SJC, CRP, and ESR), the software assigns a score (expressed as a value d) reflecting the strength of the observed differences. To assess the significance of this relationship, a value q is calculated by permutations of the measurements to estimate the percentage of parameters identified by chance, the false discovery rate (FDR). Using SJC, CRP, or ESR as quantitative response parameters, d>2 (indicating that the strength of the association between the histological input parameter and the disease activity outcome parameter was at least twice the expected value) and q<0.10 (corresponding to an α error of less than 10% in classical statistics or, in other words, indicating that the observed associations had a 90% chance of being real) were considered significant.

Finally, histological parameters that not only are correlated with disease activity but also contribute significantly to the prediction of the disease activity in individual samples were identified by PAM (predictive analysis of microarray) software [23]. Whereas SAM is intended to identify significant differences between groups of samples, PAM is intended to identify those parameters that are most useful in predicting the outcome (in this case, disease activity) in individual samples and to combine those parameters in an optimal multiparameter algorithm to classify single samples or patients.

## Results

### Comparative histopathology of SpA and RA

**Figure 1:**
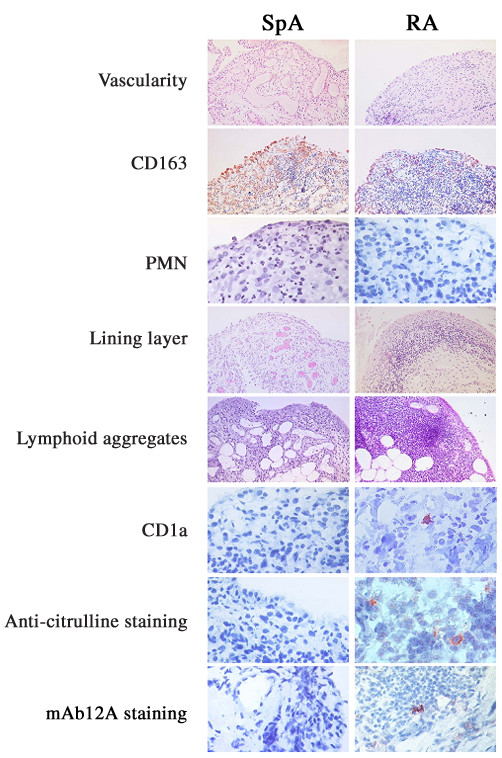
Distinct synovial features in spondyloarthropathy (SpA) and rheumatoid arthritis (RA). Vascularity, CD163^+ ^macrophages, and polymorphonuclear leukocytes (PMNs) were significantly increased in SpA, whereas lining-layer hyperplasia, lymphoid aggregates, CD1a^+ ^dendritic cells, intracellular citrullinated proteins (detected by anticitrulline staining), and MHC–HC gp39 complexes (detected by staining with monoclonal antibody (mAb) 12A) were higher in RA.

The scores for the histopathological features of the synovial membrane in SpA and RA are given in Table 2. There was significantly higher vascularity (*P *= 0.013), lining CD163 (*P *< 0.001), and sublining CD163 (*P *= 0.003) in SpA than in RA. There was also a trend towards an increase of PMNs in SpA (*P *= 0.062). In contrast, there was a significantly lower score in SpA versus RA for lining-layer thickness (*P *= 0.032), CD1a (*P *= 0.009), lymphoid aggregates (*P *= 0.029), anticitrulline staining (*P *< 0.001), and mAb 12A staining (*P *< 0.001). In contrast with CD163, no differences were found for the pan-macrophage marker CD68 in the lining or sublining layer. The number of plasma cells, which were found in 32 of the 82 SpA samples and 40 of the 86 RA samples, was also not different between the two diseases. Although the mean age of the patients was higher in the RA cohort (56.2 ± 14.9 years) than in the SpA cohort (42.6 ± 13.3 years), none of the differentiating parameters was related to age, excluding the possibility that the difference in age could have induced a systematic bias in the comparison. These findings, which are illustrated in Fig. 1, are in agreement with previous observations [1-4] and indicate that the patient cohorts used in the present study are representative of the full-blown SpA or RA synovial histopathology.

### Histopathological heterogeneity within SpA

**Figure 2:**
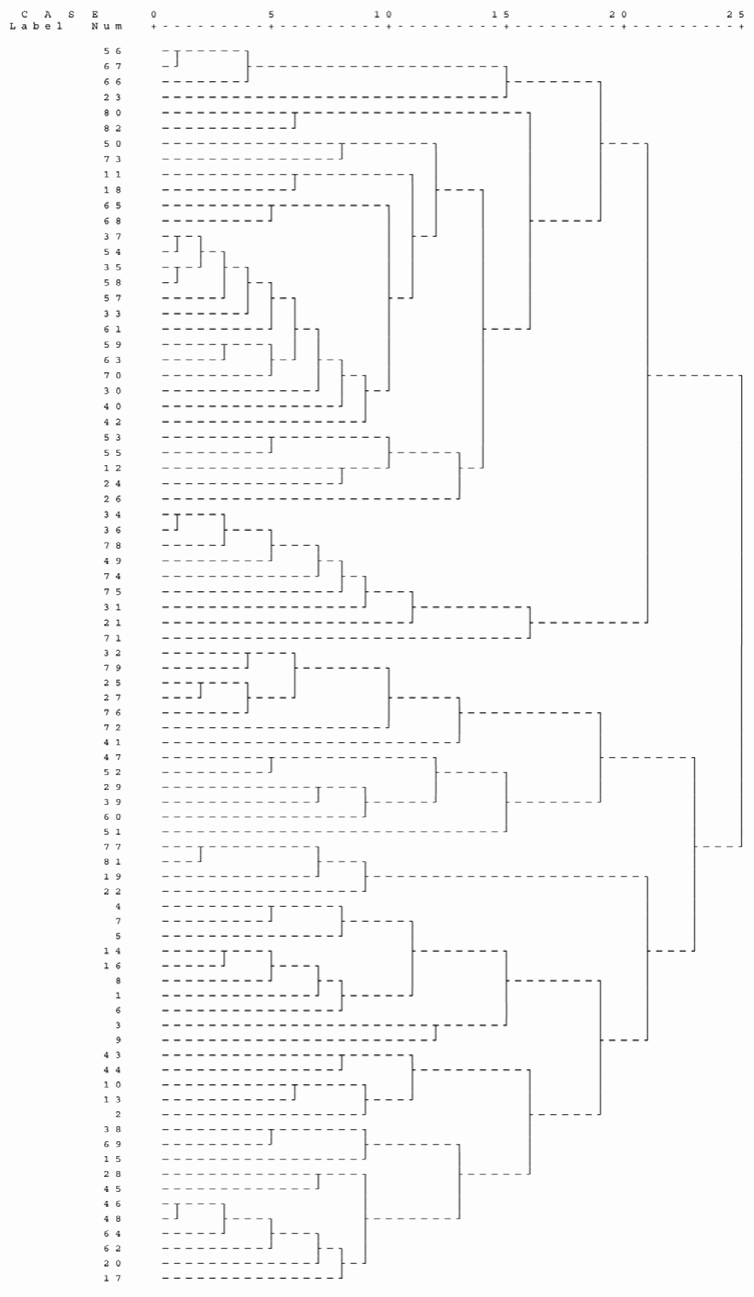
Unsupervised clustering analysis using synovial histopathological parameters identified two main clusters within the spondyloarthropathy cohort (*n *= 82). The dendrogram represents the 82 spondylarthropathy cases on the *y*-axis, classified according to their similarity for the histological parameters. The degree of similarity is represented as rescaled distance on the *x*-axis: when two samples are closely similar the distance will be small, whereas a rescaled distance of 25 represents a high degree of histological difference.

**Figure 3:**
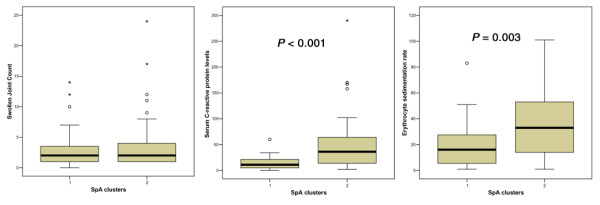
Unsupervised clustering analysis using synovial histopathological parameters identified two main clusters within the spondyloarthropathy (SpA) cohort (*n *= 82). Whereas there were no differences for the swollen jount count between the two clusters, cluster 2 was characterized by significantly higher serum C-reactive protein concentrations (mg/L) and erythrocyte sedimentation rate (mm/hour). Data are represented as box–whisker plots, with median, 25th to 75th percentile, and 5th to 95th percentile. Comparisons were performed with the Mann–Whitney *U *test.

With the exception of anticitrulline and mAb 12A staining, which were found almost exclusively in RA, all investigated histopathological parameters showed a wide range of scores within the SpA group, reflecting wide interindividual variability. Therefore, we next tried to identify specific SpA subgroups by combining the different histological features in a multiparameter model using clustering analysis. Unsupervised analysis yielded two main clusters within SpA, consisting of 39 and 43 samples (Fig. 2). Although there were slightly more AS samples in cluster 2, the different SpA subtypes were found both in cluster 1 (4 AS, 14 USpA, 16 PsA, 4 inflammatory bowel disease, 1 reactive arthritis) and in cluster 2 (15 AS, 10 USpA, 17 PsA, 1 reactive arthritis), confirming that synovial histopathology is not basically different between SpA subtypes. The mean duration of disease (5.4 ± 5.0 versus 5.7 ± 5.9 years, respectively), the use of DMARDs (in 10 of 39 versus 13 of 43), and the mean SJC (3.1 ± 3.3 versus 3.8 ± 4.7, respectively) were not different between cluster 1 and cluster 2. In contrast, both serum CRP concentrations (14 ± 12 mg/L versus 51 ± 56 mg/L; *P *< 0.001) and ESR (19 ± 17 mm/hour versus 35 ± 28 mm/hour; *P *= 0.003) were significantly lower in cluster 1 than in cluster 2 (Fig. 3), indicating that this unsupervised classification based on the synovial histopathology reflects the global disease activity. In contrast, a similar analysis of the RA cohort yielded five separate clusters, without significant differences in SJC, serum CRP concentrations, or ESR between the clusters (data not shown).

When the two clusters of the SpA cohort were compared, the cluster with higher CRP and ESR was found to show a significant increase of the following histological parameters: vascularity (*P *= 0.001), inflammatory infiltration (*P *< 0.001), lymphoid aggregates (*P *= 0.027), plasma cells (*P *= 0.001), PMNs (*P *< 0.001), CD3^+ ^lymphocytes (*P *< 0.001), and CD20^+ ^lymphocytes (*P *= 0.007).

### Relation between synovial histopathology and disease activity in SpA

Since these data suggest that synovial histopathology reflects global disease activity in SpA, we further analyzed the correlation of individual histological parameters with SJC, CRP, and ESR in the SpA cohort. In order to minimize the risk of false-positive results, only the parameters identified by both classical statistics and SAM analysis were considered as significant. As shown in more detail in Table 3 (top), lining-layer thickness, sublining CD68, and sublining CD163 were weakly but significantly correlated with the SJC in SpA. CRP concentrations correlated significantly with inflammatory infiltration, PMNs, and sublining CD163. Finally, ESR correlated with lining-layer thickness, inflammatory infiltration, PMNs, and sublining CD163. Thus, it appears that global disease activity in SpA is essentially associated with inflammatory infiltration with macrophages – especially the CD163^+ ^subset – and PMNs as well as with lining-layer hyperplasia. Although some of the correlations were relatively weak, the number of CD163^+ ^macrophages in the sublining appeared to be consistently correlated with the three different measures of disease activity, whereas inflammatory infiltration and PMNs showed a stronger correlation with the systemic inflammatory parameters.

For comparison, we performed the same analysis in the RA control group. As shown in more detail in Table 3 (bottom), no histological parameters were significantly correlated with the SJC. Serum CRP concentrations were significantly associated with CD3 and mAb 12A staining. ESR was correlated with CD3 as well as with sublining CD68 and anticitrulline staining. Globally, the correlations were weaker and less consistent in RA than in SpA.

### Supervised clustering in relation with disease activity in SpA

**Figure 4:**
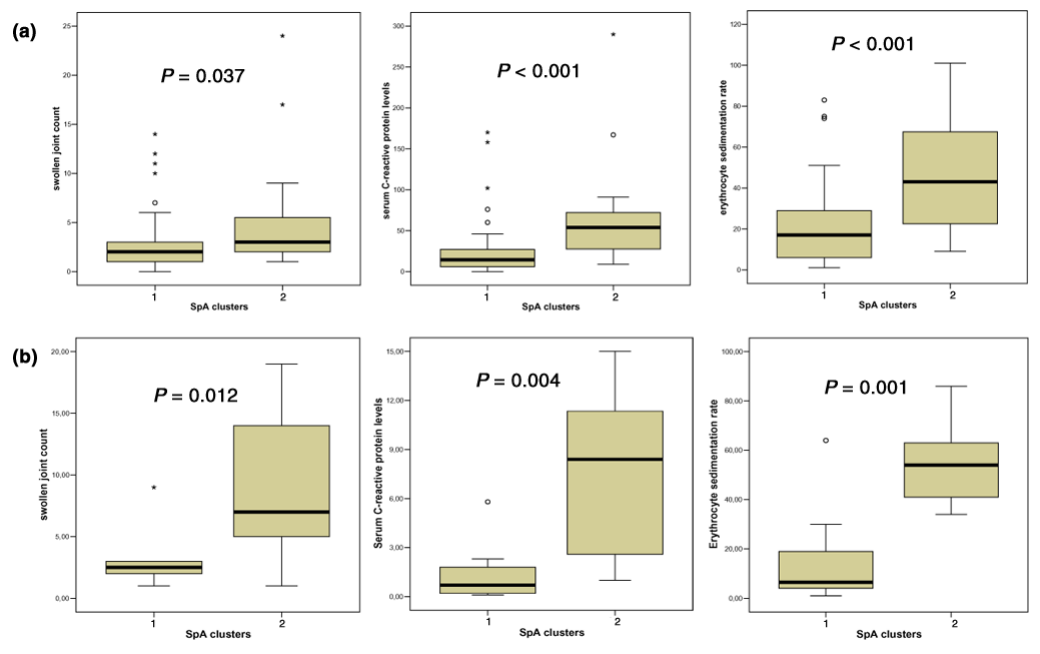
Supervised clustering analysis using the synovial histopathological parameters that were significantly associated with disease activity in spondyloarthropathy (SpA) (lining-layer hyperplasia, inflammatory infiltration, polymorphonuclear cells, sublining CD68, and sublining CD163). **(a) **Analysis in the cohort of 82 patients originally used to identify the histopathological parameters identified a cluster (cluster 2, *n *= 20 samples) which was characterized by significantly higher swollen joint count, serum C-reactive protein concentrations (mg/L), and erythrocyte sedimentation rate (mm/hour). **(b)**A similar analysis in an independent cohort of 17 patients confirmed these results by identifying a cluster of 7 patients (cluster 2) that was similarly characterized by significantly higher swollen joint count, serum C-reactive protein concentrations (mg/L), and erythrocyte sedimentation rate (mm/hour). Data are represented as box–whisker plots, with median, 25th to 75th percentile, and 5th to 95th percentile. Comparisons were performed with the Mann–Whitney *U *test.

On the basis of the previous findings, we redefined two separate clusters within the SpA cohort based not on all synovial features, but on the five synovial characteristics that were significantly associated with disease activity: lining-layer hyperplasia, inflammatory infiltration, PMNs, sublining CD68, and sublining CD163. Cluster 1 (*n *= 62) was characterized by significantly lower SJC (3.0 ± 3.2 versus 5.2 ± 5.8; *P *= 0.037), serum CRP concentrations (23 ± 32 mg/L versus 66 ± 63 mg/L; *P *< 0.001), and ESR (21 ± 19 mm/hour versus 48 ± 28 mm/hour; *P *< 0.001) than cluster 2 (*n *= 20) (Fig. 4a). Again, there were no significant differences between the two clusters with regard to the SpA subtypes (12 AS, 21 USpA, 24 PsA, 3 inflammatory bowel disease, and 2 reactive arthritis in cluster 1 versus 7 AS, 3 USpA, 9 PsA, and 1 inflammatory bowel disease in cluster 2), indicating the absence of a relation between histopathology and disease phenotypes. Moreover, there was no difference in DMARD treatment (5 of 20 versus 18 of 62) or disease duration (4.0 ± 5.3 versus 5.8 ± 5.5 years) between the two clusters. In other words, the five previously defined histological parameters were able to identify a subgroup of SpA patients with high disease activity independently of the SpA subtype, treatment, and disease duration, thereby confirming that synovial histopathology reflects not only local inflammation but also global disease activity in SpA.

In contrast, a similar analysis of the RA cohort using CD3, sublining CD68, anticitrulline, and mAb 12A as input parameters yielded two clusters (with respectively *n *= 41 and *n *= 45 samples) that were not different with regard to SJC (9.1 ± 6.3 versus 9.2 ± 7.0), serum CRP concentrations (52 ± 43 mg/L versus 64 ± 82 mg/L), or ESR (42 ± 27 mm/hour versus 40 ± 28 mm/hour).

### Confirmation of the supervised clustering analysis on an independent SpA cohort

To confirm these findings, we applied the same supervised clustering analysis based on the five previously defined histological parameters to an independent cohort of 17 SpA patients, none of whom were being treated with DMARDs. Two clusters were identified: cluster 1, consisting of 7 patients (1 with AS, 2 with PsA, 4 with USpA) and cluster 2, consisting of 10 patients (3 with AS, 3 with PsA, 4 with USpA). The mean disease duration in the two clusters was similar (10.7 ± 6.9 versus 10.8 ± 12.4 years, respectively). However, the two clusters were again significantly different with regard to SJC (9.3 ± 6.6 versus 2.9 ± 2.3; *P *= 0.012), serum CRP concentrations (75 ± 55 versus 13 ± 18 mg/L; *P *= 0.004), and ESR (55 ± 18 versus 15 ± 20 mm/hour; *P *= 0.001) (Fig. 4b). Thus, this analysis in an independent validation cohort confirms that well-defined synovial histopathological features in SpA reflect global disease activity independently of SpA subtype, disease duration, and treatment.

### Prediction of global disease activity in individual SpA samples

Since the previous data provided evidence that lining-layer hyperplasia and infiltration with PMNs and macrophage subsets are directly related to the global disease activity in SpA, we next investigated whether synovial histopathology could be a valuable surrogate marker for the prediction of disease activity in individual patients rather than in a patient cohort. Using PAM to classify the 82 SpA patients into tertiles (low, middle, and high disease activity) for respectively SJC, CRP, and ESR, the same histological parameters were identified as having the largest contribution to the predictive algorithms: inflammatory infiltration, sublining CD163, PMNs, sublining CD68, and lining CD68. However, the positive predictive value of these models was relatively poor, as shown by the correct classification of only 51%, 55%, and 49% of the samples for SJC, CRP, and ESR, respectively, compared with an a priori chance of 33%. Similarly, PAM analysis using histopathological parameters was not able to make a good prediction of samples belonging to the highest quartile for disease activity (data not shown). These data indicate that although the previously identified histological parameters are related to disease activity, the wide interindividual variability does not allow a robust prediction in single patients.

## Discussion

It has previously been shown that the synovial histopathology in inflammatory arthritis is dependent on both the disease background and the local disease activity [1,2,12,15]. When focusing on SpA patients with active synovitis of the investigated joint, however, we still observe a large heterogeneity in synovial features. In an attempt to translate these histological findings into clinically relevant patterns, we assessed whether this heterogeneity was related to the fact that SpA consists of different subtypes with distinct phenotypes. Confirming a recent report in which we found no significant differences between PsA on the one hand and AS and USpA on the other hand (unpublished data), both unsupervised and supervised clustering analysis of the present data indicated that the synovial heterogeneity is not basically associated with specific SpA phenotypes.

In contrast, the present study reveals that this heterogeneity directly reflects the global disease activity in SpA. Considering that there are no validated global disease parameters for SpA as a whole, the present study used SJC, serum CRP concentrations, and ESR as clinical outcomes. Although these parameters are not elevated in all patients with active SpA, several studies have shown that peripheral arthritis as well as systemic inflammatory parameters are characteristics of severe disease [24,25]. In this context, the present finding that local synovial features are correlated with systemic inflammatory parameters such as CRP and ESR strengthens the concept that peripheral synovitis, although not present in all SpA patients, contributes significantly to disease severity.

Further analysis revealed that not all synovial features were correlated with disease activity. Both classical statistics and SAM analysis demonstrated a correlation with lining-layer hyperplasia and, more consistently, inflammatory infiltration by CD163^+ ^macrophages and PMNs. We previously shown that these CD163^+ ^macrophages, but not the overall number of CD68^+ ^macrophages, are increased in SpA synovitis and play a specific role in the disease pathogenesis [2,26]. In contrast, neither synovial hypervascularity, which is clearly increased in SpA versus RA and contributes to diagnostic classification [1,7], nor lymphocyte-related characteristics (CD3, CD20, plasma cells, lymphoid aggregates) were associated with SJC, CRP, or ESR. This was further confirmed by a supervised clustering analysis that could even better identify a high-disease-activity group on the basis of only five synovial features, both in the first SpA cohort that was used to identify these features and, most importantly, in a completely independent SpA cohort. Both the previous reports and the present study pointed towards the increased presence of CD163^+ ^macrophages and PMNs in SpA versus RA synovitis [2,27]. Moreover, in the RA control group the global disease activity parameters correlated not with these features, but with lymphocyte-related characteristics such as CD3 and putative B- and T-cell autoantigens in RA (intracellular citrullinated proteins and MHC–HC gp39 complexes) [3,4]. Although certainly not excluding a secondary effector function for lymphocytes in SpA or for macrophages in RA synovitis, these findings point towards distinct pathogenetic mechanisms in the two diseases and fit well with the hypothesis that SpA synovitis is primarly driven by innate immune cells with secondary alterations in lymphocyte activation and functions [28].

Independently of these pathogenetic considerations, the present data also raise the question of the value of synovial histopathology as a biomarker in SpA. Despite the fact that lining-layer hyperplasia and inflammatory infiltration with CD163^+ ^macrophages and PMNs reflected the global disease activity when cohorts of patients were analyzed, multiparameter models based on synovial histopathology turned out to be relatively poor predictors of disease severity in individual patients. This finding is not totally unexpected in view of the wide variability of individual values for both histology and disease activity and the broad overlap between different clusters in the previous analyses.

Several factors could play a role in this wide individual variability. Firstly, treatment with DMARDs might be a confounding factor. However, the facts that most SpA patients of the first cohort had been given no treatment at all or were being treated exclusively with nonsteroidal antirheumatic drugs, that the clustering analysis was not influenced by treatment, and that the clustering was confirmed in an independent cohort without DMARD treatment are in accord with previous data showing that DMARDs did not bias the synovial histopathology in patients with persistent, refractory peripheral synovitis (unpublished data). Moreover, even after exclusion of the DMARD-treated patients, the prediction models performed poorly (data not shown). Secondly, the present study used a semiquantitatve scoring system for the histopathology, whereas the previously mentioned RA studies used digital image analysis [16]. Whereas semiquantitative scoring has been shown in multiple studies to be robust and reproducible, it is less sensitive to change than digital image analysis and might thus underestimate small variations [29]. Thirdly, recent data obtained with microarrays indicated clearly that setting up profiles using multiple parameters can compensate for the relative lack of precision and the variability of individual parameters [30]. As we have already demonstrated the added value of combining different histopathological features in multiparameter models for diagnostic classification of inflammatory arthritis [7,8], the same might apply to the use of synovial histology as a surrogate marker for global disease activity.

In this context, early–phase, randomized clinical trials in SpA might be of particular interest for the use of synovial histopathology as a biomarker. With the availibility of powerful new treatments such as TNF-α blockers it becomes increasingly important to obtain as much paraclinical and biological information as possible in small patient cohorts early in the clinical development of new drugs. Moreover, in such trials, the emphasis is on groups with uniform treatment schedules rather than on individual patients, and different biological measurements (such as histology, mRNA expression levels, serum protein concentrations) can be combined in multiparameter algorithms, thus overcoming the previously mentioned caveats. In RA, it has recently been demonstrated that the number of sublining CD68^+ ^macrophages is a sensitive surrogate marker for response to therapy [16], even if this feature was only found to correlate with ESR in RA in the present cross-sectional study and was clearly less robust than the previously discussed SpA parameters. Since the correlations between global disease activity and synovial histopathology of a single joint were consistently stronger in SpA than in RA and since previous studies showed a histopathological response to targeted therapies in SpA and more specifically a decrease of macrophage subsets and PMNs [17,18,31-33], the data presented here warrant further prospective and longitudinal analysis of synovial histopathology as a surrogate marker in the evaluation of new, targeted therapies for SpA.

## Conclusion

The data presented indicate that inflammatory infiltration of the synovium with CD163^+ ^macrophages and PMNs as well as lining-layer hyperplasia reflect global disease activity in SpA, independently of the SpA subtype. These data support a prominent role for innate immune cells in SpA synovitis and warrant further evaluation of synovial histopathology as a surrogate marker in early-phase therapeutic trials in SpA.

## Abbreviations

AS = ankylosing spondylitis; CRP = C-reactive protein; DMARD = disease-modifying antirheumatic drug; ESR = erythrocyte sedimentation rate; H & E = hematoxylin and eosin; IL = interleukin; mAb = monoclonal antibody; MHC = major histocompatibility complex; PAM = predictive analysis of microarray; PMN = polymorphonuclear leukocyte; PsA = psoriatic arthritis; RA = rheumatoid arthritis; SAM = significance analysis of microarray; SJC = swollen joint count; SpA = spondyloarthropathy; TNF = tumor necrosis factor; USpA = undifferentiated spondyloarthropathy.

## Competing interests

Annemieke M Boots is employed by Organon NV, Oss, The Netherlands.

## Authors' contributions

DB, EMV, and FDK designed the study. DB, EK, and LDR sample and analyzed the synovial tissues. HM selected the patients. DB collected and analyzed the data. mAb 12A was provided by AMB. DB, LDR, and AMB prepared the manuscript, and EMV, HM, and FDK reviewed it. All authors read and approved the final manuscript.

## Figures and Tables

**Table 1:** Histopathological features and scoring systems used to evaluate synovial inflammation

Feature	Scoring system
*Assessed by histology*	
Lining-layer hyperplasia	Semiquantitative
Degree of vascularity	Semiquantitative
Inflammatory infiltration	Semiquantitative
Lymphoid aggregates	Present or absent
Plasma cells	Semiquantitative
Polymorphonuclear leukocytes	Semiquantitative
*Assessed by immunohistochemistry*	
CD1a	Present or absent
CD3	Semiquantitative
CD20	Semiquantitative
CD68 lining layer	Semiquantitative
CD68 sublining layer	Semiquantitative
CD163 lining layer	Semiquantitative
CD163 sublining layer	Semiquantitative
Intracellular citrullinated peptides	Present or absent
MHC class II–HC gp39 peptide complex, recognized by mAb 12A	Present or absent

**Table 2:** Synovial histopathology in spondyloarthropathy and rheumatoid arthritis

Feature	Spondyloarthropathy^a^	Rheumatoid arthritis^a^
Lining-layer thickness	1 (1–3)*	1.5 (1–3)*
Vascularity	2 (0–3)*	1.5 (0–3)*
Inflammatory infiltration	1.5 (0–3)	2 (0–3)
Plasma cells	0 (0–3)	0 (0–3)
Polymorphonuclear leukocytes	0 (0–3)	0 (0–2.5)
CD3	1.5 (0–3)	2 (0–3)
CD20	1 (0–3)	1.5 (0–3)
CD68 in lining layer	1 (0–3)	1 (0–3)
CD68 in sublining layer	0.5 (0–3)	1 (0–3)
CD163 in lining layer	1.5 (0–3)*	0.5 (0–3)*
CD163 in sublining layer	1.5 (0–3)*	0.5 (0–3)*
Lymphoid aggregates	17/65*	32/54*
CD1a	18/64*	36/50*
Anticitrulline staining	0/82*	27/59*
mAb 12A staining	1/81*	28/58*

^a^Results are expressed as median (range) for semiquantitative scores and present/absent for dichotomous parameters. As indictated in Materials and methods, stained sections were scored by two blinded observers using a semiquantitative scale from 0 (lowest expression) to 3 (highest level of expression). This scale was calibrated for each marker separately and the mean of the two scores was used.

*P < 0.05.

**Table 3:** Association of single histopathological features with three measures of disease activity in spondyloarthropathy (top) and in rheumatoid arthritis (bottom)

Histological feature	SJC		CRP		ESR	
			
	Correlation^a^	SAM	Correlation^a^	SAM	Correlation^a^	SAM
***In spondylarthropathy***						
Lining-layer thickness	0.220	2.16	0.242	NS	0.240	2.30
Vascularity	NS	NS	NS	NS	NS	NS
Inflammatory infiltration	NS	NS	0.472	2.60	0.336	2.34
Plasma cells	NS	NS	NS	NS	NS	NS
PMNs	NS	NS	0.393	2.15	0.280	2.58
CD3	NS	NS	0.236	NS	NS	NS
CD20	NS	NS	NS	NS	NS	NS
Lining CD68	NS	NS	-0.510	NS	-0.372	NS
Sublining CD68	0.278	3.14	NS	NS	NS	NS
Lining CD163	NS	NS	NS	NS	NS	NS
Sublining CD163	0.281	2.14	0.249	2.06	0.299	3.18
Lymphoid aggregates	NS	2.23	NS	NS	NS	NS
CD1a	NS	NS	NS	NS	NS	NS
Anticitrulline staining	NS	NS	NS	NS	NS	NS
mAb 12A staining	NS	NS	NS	NS	NS	NS
***In rheumatoid arthritis***						
Lining-layer thickness	0.222	NS	NS	NS	NS	NS
Vascularity	NS	NS	NS	NS	-0.235	NS
Inflammatory infiltration	NS	NS	NS	NS	NS	NS
Plasma cells	NS	NS	NS	NS	0.212	NS
PMNs	-0.255	NS	NS	NS	NS	NS
CD3	NS	NS	0.297	2.02	0.334	2.49
CD20	NS	NS	NS	NS	NS	NS
Lining CD68	NS	NS	-0.227	NS	NS	NS
Sublining CD68	NS	NS	NS	NS	0.304	2.31
Lining CD163	NS	NS	NS	NS	NS	NS
Sublining CD163	NS	NS	NS	NS	NS	NS
Lymphoid aggregates	NS	NS	NS	NS	NS	NS
CD1a	NS	NS	NS	NS	NS	NS
Anticitrulline staining	NS	NS	NS	NS	*P *= 0.004	2.84
mAb 12A	NS	NS	*P *= 0.035	2.00	NS	NS

Only significant associations are shown. ^a^Correlations were calculated either by classicial statistics (results are expressed as correlation coefficient; for dichotomous parameters differences between the positive and negative group are expressed as *P *on Student's unpaired *t*-test) or by SAM (significance analysis of microarray) (for which results are expressed as d values). CRP, serum C-reactive protein; ESR, erythrocyte sedimentation rate; NS, not significant; PMNs, polymorphonuclear leukocytes; SJC, swollen joint count.
